# Radiotherapy in Italian media: (mis)information, patients’ perception and medical career choices

**DOI:** 10.1007/s11547-025-02159-9

**Published:** 2025-12-03

**Authors:** Federico Gagliardi, Emma D’Ippolito, Roberta Grassi, Angelo Sangiovanni, Vittorio Salvatore Menditti, Dino Rubini, Paolo Gallo, Luca D’Ambrosio, Luca Boldrini, Viola Salvestrini, Isacco Desideri, Francesca De Felice, Giuseppe Carlo Iorio, Antonio Piras, Luca Nicosia, Valerio Nardone

**Affiliations:** 1 Department of Precision Medicine, University of Campania “L. Vanvitelli”, Via De Crecchio 7, 80138 Naples, Italy; 2 Radiation Oncology, Fondazione Policlinico Universitario A. Gemelli, IRCCS, Largo Agostino Gemelli, 00168 Rome, Italy; 3 Università Degli Studi Di Firenze, Azienda Ospedaliero Universitaria Careggi, Florence, Italy; 4 apienza” Università Di Roma, Azienda Ospedaliero Universitaria Policlinico Umberto I, Rome, Italy; 5 Azienda Ospedaliera Universitaria Città Della Salute E Della Scienza Di Torino, Turin, Italy; 6 Villa Santa Teresa –Università di Palermo, Palermo, Italy; 7 Advanced Radiation Oncology Department, IRCCS Sacro Cuore Don Calabria Hospital, Cancer Care Center, Verona, Italy

**Keywords:** Radiation oncology, Media sentiment, Public perception

## Abstract

**Aims:**

The aim of this study is to analyze how radiotherapy (RT) is perceived and portrayed by Italian media and to determine whether there is any bias or misinformation about. The study will also assess the influence of these perceptions on patients’ and medical students’ decisions to specialize in radiotherapy.

**Methods:**

A comprehensive review was conducted of 436 articles published in “Corriere della Sera” between 1895 and 2023, using keywords such as "radiotherapy" and "radiation." The articles were classified into positive, neutral, and negative categories, and the dominant themes and trends were analyzed.

**Results:**

Articles on radiotherapy (RT) have significantly increased since year 2000, with a notable rise in negative publications focused on toxicities and alleged malpractice.

Out of 436 articles, 74 were negative, with this trend growing in recent years, emphasizing risks over benefits in media coverage of RT.

**Conclusions:**

The influence of media on public perception of RT is significant and influences clinical and therapeutic decisions. It is essential that the RT community continues working with media and communication professionals to promote accurate information about the benefits and advances of RT for the patients and for healthcare professionals.

Advances in Knowledge.

This study highlights the importance of accurate media portrayal of RT to improve public understanding of its benefits. Collaboration between radiation oncologists and media can help disseminate positive outcomes and dispel harmful myths to ensure a balanced and informed perception of RT.

## Introduction

Radiotherapy (RT) is a fundamental treatment in oncology. The technique aims to deliver radiation precisely to the tumor while preserving surrounding healthy tissue, in a strictly personalized manner [1]. It is estimated that at least 50% of cancer patients will undergo a course of RT at some point and in many cases, it may be prescribed as primary curative therapy [2].

The development of new technologies has led to improved patient safety and better results in radiotherapy treatments. This progress has introduced a new paradigm for more personalized radiotherapy, which translates into targeted, more effective, and safer treatments for patients [3].

Numerous clinical trials have demonstrated that RT can offer outcomes comparable or even superior to surgical treatments in selected patient populations. This underlines its curative potential and relevance in modern oncology [4, 5].

Despite these beneficial innovations, some people still have concerns about radiation exposure associated with RT treatments. These concerns may arise from a lack of understanding of the risks and benefits of RT, as well as fears related to the nature of radiation itself [6].

Education and counseling provided by medical professionals can be crucial in addressing these concerns and ensuring that patients have a complete understanding of the benefits of treatments and their path to recovery.

It is important to note that medical information usually comes not only from doctors, but also from media and other sources, and a biased or superficial exposure can negatively influence patients’ perceptions of their disease and treatment options [7].

In recent times, there has been an increase in negative media sentiment toward radiation oncology and a reduced recognition of this discipline even in specialized press. These conditions potentially impact public perception worldwide with serious consequences in terms of access to cures and aware and fair doctor–patient relationship [8].

Accurate and robust communication on RT is therefore essential to raise awareness and to ensure that patients have appropriate knowledge of the treatment options, also influencing healthcare providers and future practitioners. In today’s healthcare landscape, where the figure of the radiation oncologist often remains poorly known, deepening the potential of radiotherapy can significantly influence young doctors in their choice of post-graduate specialization. This approach would not only enrich their cultural background but also highlight the real benefits of radiation treatments, giving them the necessary tools to make a more informed and conscious choice.

The current shortage of radiation oncologists will have significant implications for patient health and cancer therapy management that could put European National Health Systems in trouble in the very near future[9].

### Aim of the study

In an era where the media has a significant influence on public opinion, it is crucial to analyze their content and better understand their impact on the perception of the different scientific and biomedical disciplines. This study aims to investigate how RT is perceived by Italian media and whether there are widespread prejudices or misinformation on the subject. This information could be useful in educating the public about the importance and effectiveness of RT in cancer treatment and in promoting a more balanced and accurate representation of the topic in the media. Furthermore, it is worth considering how this reputation could potentially influence the decision-making process of Italian medical students when it comes to choosing their area of specialization, which currently represents a real emergency for the Italian national system.

Recent national data have revealed that 55.3% of radiation oncology residency positions in Italy have remained unfilled or been abandoned since 2016, rising to a dramatic 90% vacancy rate in 2023.

This phenomenon is further compounded by inadequate RT exposure during undergraduate training, limited financial incentives, and negative societal perceptions. This shortage portends a looming crisis for the Italian National Health System, imperiling its capacity to meet the escalating demand for RT services [9].

## Materials and methods

### Data collection

The Corriere della Sera, Italy’s leading newspaper, founded in 1876, provides free access to its extensive digital archives spanning over a century. For this study, we analyzed articles published between 1 January 1895 and 31 December 2023, focusing on publications relevant to radiation oncology. This timeframe was selected to encompass all developments in radiation-related sciences since the discovery of X-rays in 1895.

To identify relevant publications, a keyword-based search was conducted in the archives available on the newspaper’s official website. Specifically, we queried articles containing the Italian terms “radiazione” (radiation) or “radioterapia” (radiotherapy) in either the title or the main text.

### Data selection

To ensure the dataset’s relevance to the field of radiation oncology, a systematic and transparent two-step filtering process was employed:

- Relevance filtering: The dataset was refined to include only articles containing oncology-related terms such as “paziente*” (patient), “radioterap*” (radiotherapy), “oncolog*” (oncology), or “cancro” (cancer). These terms were chosen to focus specifically on content explicitly related to clinical applications and treatment aspects of radiation oncology.
- Independent Manual Review: To enhance the reliability of the selection process, two independent researchers (FG and DR) reviewed the articles that passed the keyword filter. Each researcher independently assessed the relevance of the articles to ensure they provided insights into radiation oncology advancements, patient care, or treatment methodologies. Any discrepancies between the researchers’ selections were resolved through discussion and consensus with a third author (VN).

This systematic and collaborative approach ensured that the final dataset was both comprehensive and of high quality, providing a robust basis for the analysis of historical trends and developments in radiation oncology as documented in Corriere della Sera.

### Data categorization

Following the careful review and selection articles specifically related to medical applications of radiation therapy (RT), we proceeded to classify the articles into ten distinct categories based on their overarching focus and sentiment toward RT[8]. This categorization aimed to provide a nuanced understanding of the historical and thematic perspectives documented in the analyzed content.

The ten categories were divided by sentiment into:

- Positive Perspective (3 categories): Articles emphasizing advancements, successful treatments, innovations in RT technologies, and its potential for improving patient outcomes.
- Neutral Perspective (2 categories): Articles presenting balanced or informational content, including technical descriptions, procedural explanations, or reports on RT usage without clear advocacy or criticism.
- Negative Perspective (5 categories): Articles highlighting risks, controversies, societal concerns, or negative outcomes associated with RT, such as potential harm to patients, ethical debates, or public fears about radiation exposure.

In addition to the categories identified by previous authors, we have added a new category (Category 11) for articles that address the shortage of radiation oncology specialists and residents. The purpose of this category is to highlight the challenges caused by the inadequate number of radiation oncology specialists and trainees and its potential impact on patient care and treatment outcomes in our country.

This category of articles covers topics such as staff shortage, recruitment challenges, and the necessity to expand training programs to meet the increasing demand for RT services.

This systematic categorization allowed for the identification of common themes, such as the portrayal of RT in public discourse, the evolution of its reputation over time, and shifts in its perception in the medical community.

## Results

The initial keyword-based search for articles containing the terms “radiation” or “radiotherapy” in the titles or texts yielded a dataset of 1,682 publications. After a careful review and selection process, we included 436 articles that specifically pertained to the medical applications of radiation therapy (RT).

**Fig. 1:**
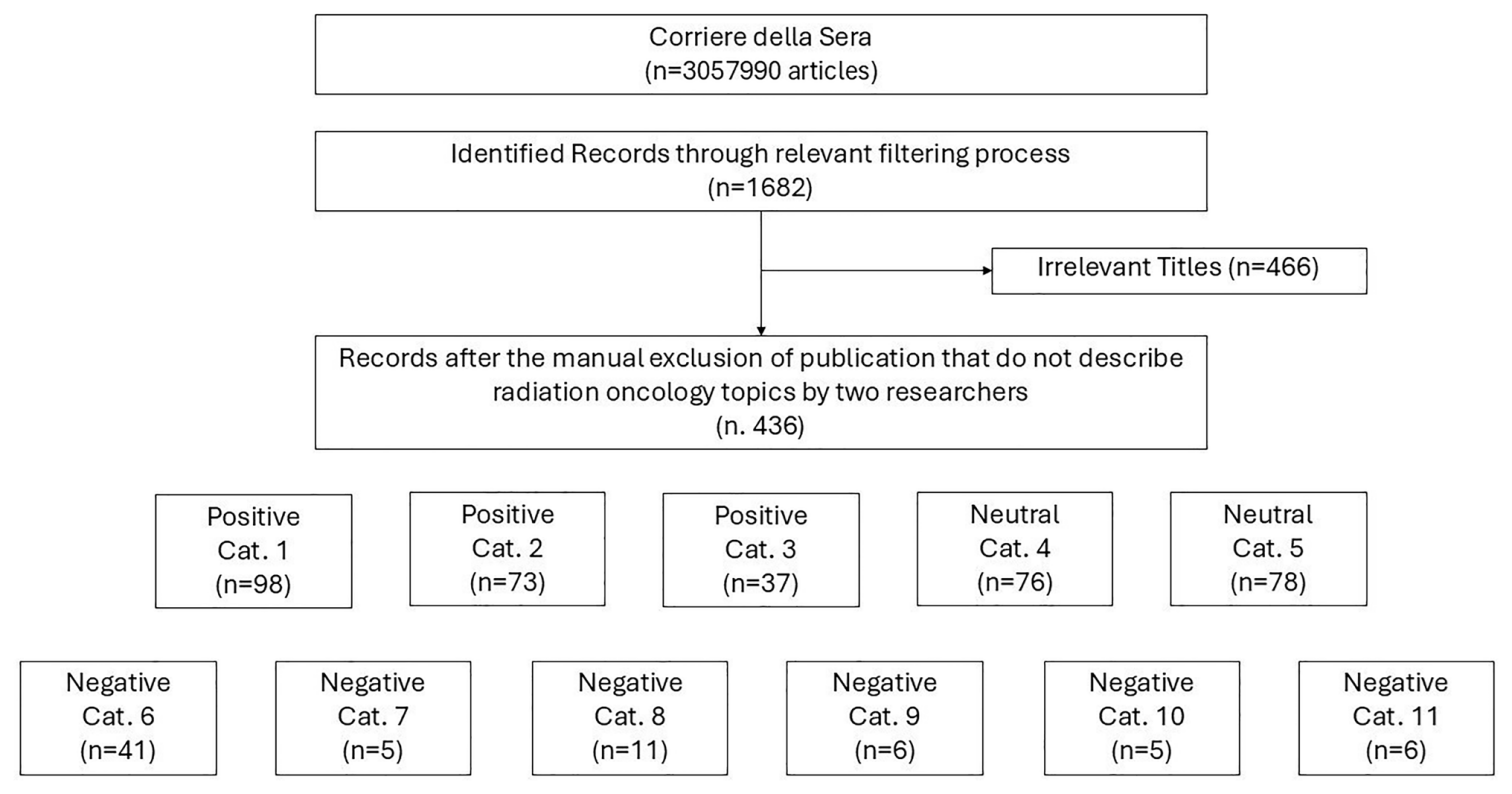
Articles selection workflow

Figure 1 shows the workflow used in the selection of articles. These articles span the period from 1904 to 2023, reflecting over a century of discourse and developments related to RT in Corriere della Sera. The final dataset provides a comprehensive foundation for analyzing the historical evolution, societal perceptions, and challenges associated with RT within this timeframe. Out of the articles analyzed, 286 dealt with topics related to Italy, while nine focused on the USA, ten on Europe, three on Asia, two on Africa, and one on Brazil.

Following the model of other authors, our analysis identified ten main categories that summarize the media issues related to radiotherapy [8].

These categories were classified as negative (five), positive (three), and neutral (two), as shown in Table 1.

**Table 1:** Media Coverage Sentiment Analysis of Radiotherapy in “Corriere della Sera” (1895–2023) This table categorizes and quantifies the media coverage of radiotherapy (RT) in “Corriere della Sera” from 1895 to 2023, highlighting the distribution of positive, neutral, and negative portrayals

Sentiment	Category	Description	Count
Positive	1	RT treatment success	98
	2	Technology innovations in RT	73
	3	Superiority and safety of RT	37
Neutral	4	Celebrities’ RT treatment	76
	5	Implementing RT in hospitals	78
Negative	6	Risks and toxicity of RT	41
	7	Equipment malfunctions and errors	5
	8	Treatment inefficacy and relapse	11
	9	RT as treatment with limited use, avoidance in treatment	6
	10	Sub-optimal RT practices	5

Of the 436 articles selected for analysis, 74 (17%) were classified as “negative” (categories 6–11), 154 (35%) were classified as “neutral,” and 208 (48%) were classified as “positive.”

Out of the various categories of negative articles, it seems that the largest number of articles is related to category 6 (41 articles − 9%), which deals with toxicities and risks associated with RT treatments.

Analyzing the temporal distribution, we observe a general increase in the number of articles addressing radiotherapy starting in the 1960s, reaching a peak during the decade 2001–2010. This is followed by a notable decline in publications from 2011 onward, with a slight uptick in the number of articles published after 2021.

There is a complete absence of publications between 1936 and 1948, and the number of articles remains low throughout the twentieth century. A significant shift occurs only after 2000, when 315 articles (72% of the 436 selected) were published. This trend aligns with the overall increase in the number of articles published in Corriere, with 74% of all articles appearing after 2000.

In terms of the ratio of articles reflecting negative sentiments toward radiotherapy, the highest relative proportion is observed in the 1950s. Although this proportion declined in subsequent decades, articles with negative sentiment have consistently been present over time.

Until 2000, articles mainly focused on the creation of new RT departments (category 5: 23 out of 126 articles) and the effectiveness of RT (category 1: 31 out of 126 articles).

Shifting our attention to the oncological diseases that have attracted the most media attention, we note that prostate cancer (18 articles) and breast cancer (26 articles) were the major players. In contrast, only 11 articles described applications of radiotherapy in non-oncological areas.

**Fig. 2:**
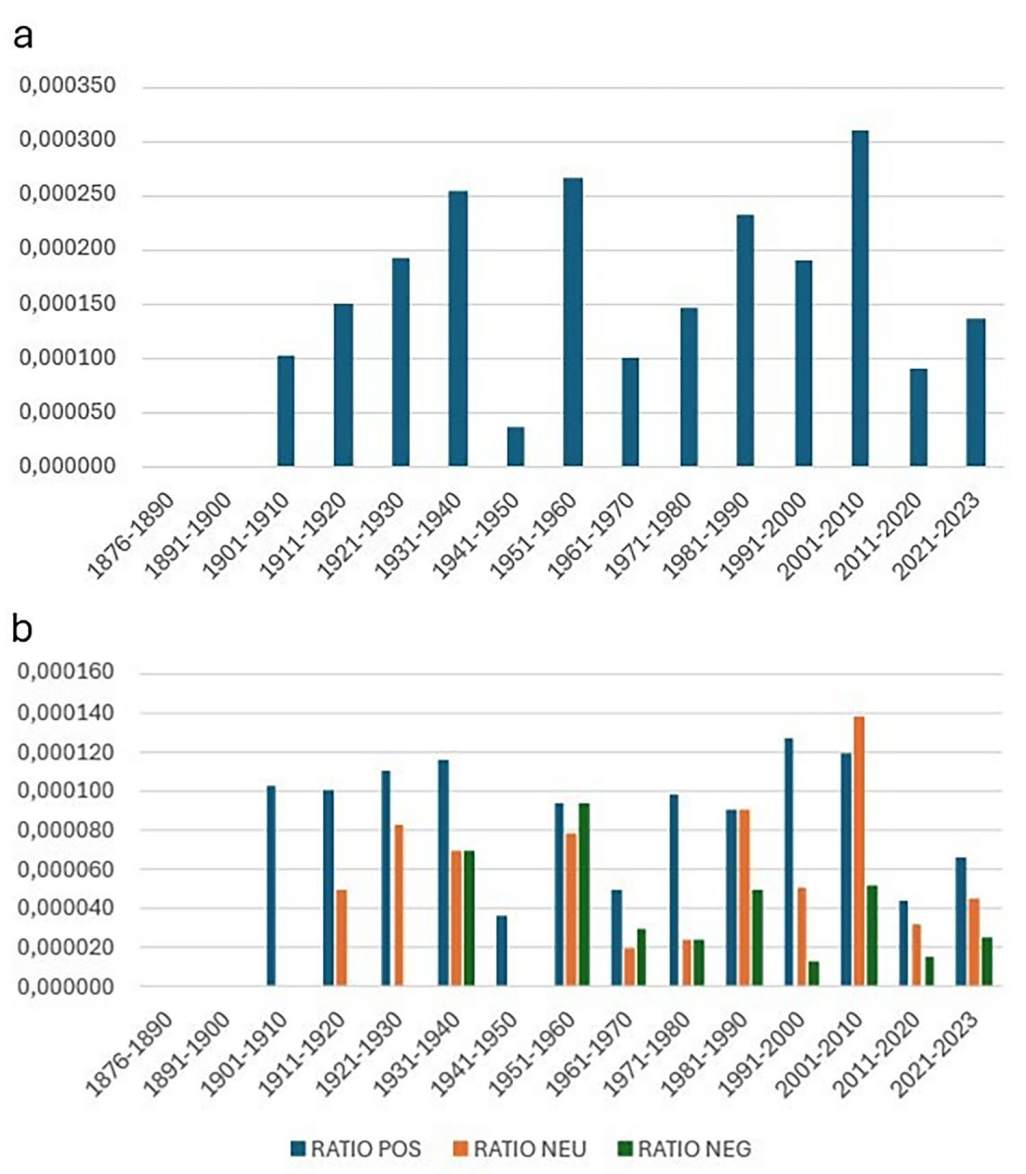
**a** the trend in the total ratio of articles dealing with radiotherapy over time, broken down by intervals of years. The trend shows significant fluctuations, with peaks in periods such as 2001–2010 and a clear decline in subsequent years, up to the period 2021–2023. **b** Time course of reports (ratios) of articles on radiotherapy divided by sentiment: positive (RATIO POS), neutral (RATIO NEU), and negative (RATIO NEG)

Figure 2a and b illustrates the trend in media interest in radiotherapy, measured by the ratio of positive, neutral, and negative articles published over time.

Despite the presence of a range of sentiments, positive articles have been found to predominate over time. The general decline in ratios observed during the period 2021–2023 is indicative of a decrease in the overall attention devoted to radiotherapy in the media.

During the final year of the period considered, a new trend emerged regarding the shortage of radiotherapy trainees, with five articles published in 2023 alone. This phenomenon indicates a growing consciousness of the problems associated with the training and availability of radiotherapy trainees in Italy.

## Discussion

The mass media have a considerable impact on people’s way of thinking. Indeed, it is crucial to pay attention to the messages and patterns they transmit, as they have the power to shape and orient public opinion, even about very advanced knowledge domains such us biomedical sciences in general and radiation oncology in particular. This is generally determined by selecting and presenting only some news to influence the public agenda and general thought.

The power of the media to influence public opinion is such that diseases that occur frequently in the media and TV series are perceived as more serious than those that are rarely found in programs but are clearly more difficult to manage [10].

This potential enables the media to play a significant role in influencing healthcare intervention utilization by promoting a specific medical specialty while overlooking others [11].

In the analysis of the articles published in the Corriere della Sera, it was observed that in the early days of RT, there was a lack of clarity regarding its true definition, which was often confused with nuclear medicine therapies.

Despite the advent of a new branch of medicine, articles about this specialty remained scarce in the early 1900s. There was a notable absence of articles from 1936 to 1948, a period when tumors were not a prominent focus of news topics not being the primary cause of death and with a specific radiation taboo after the events of the second world war [12].

Among the pathologies discussed in the context of RT, certain diseases such as prostate and breast cancer were the most treated, probably due to their high incidence which inevitably attracted media attention.

As scientific knowledge has increased, inevitably the practical applications have also increased and this has led to the introduction of radiotherapy even intraoperatively, we are therefore talking about IORT, and the creation of new technologies such as Gamma Knife.

Another topic covered is the use of RT in non-oncological settings, with 11 dedicated articles, mostly published before 1990. However, despite the increasing popularity of these applications, their absence in recent articles is noteworthy[13].

Another interesting aspect that we can highlight is the presence of articles describing the experiences of celebrities (category 4), with a total of no less than 76 articles, so numerous probably due to the media power of the personalities mentioned, who inevitably easily attract readers.

Here’s a revised and expanded version of the section, incorporating a deeper discussion about the total number of articles, negative sentiments, and the importance of surveillance:

Another noteworthy aspect is the decline in the relative number of articles addressing radiotherapy (RT) since 2011, despite the substantial technological advancements in the field over the past decades. This decrease is significant because it coincides with a period marked by major innovations in radiation oncology, which should ideally garner greater media attention due to their potential impact on patient care. [14]The decrease in coverage is concerning not only in terms of the overall visibility of RT in Italian media but also in relation to the persistent presence of negative sentiment articles. While the ratio of negative articles was highest in the 1950s, negative sentiment has continued to be a feature of RT-related publications, even as the field has evolved, reflecting a troubling trend in the media’s portrayal of the discipline during a time of rapid progress. These negative articles, even if limited in number, may disproportionately shape public perceptions and influence patient decisions, potentially undermining trust in RT as a treatment modality. It is essential for the scientific community, particularly radiation oncologists, to remain vigilant regarding the discipline’s representation in the media. Monitoring trends in the total number of articles published and actively addressing negative coverage is critical. Efforts to ensure accurate, balanced, and positive portrayals of RT advancements are vital to improving public understanding, fostering trust, and encouraging informed decision-making by patients. In this context, initiatives to increase the visibility of RT in Italian media should be prioritized. These could include public awareness campaigns, collaborations with journalists to highlight breakthroughs in the field, and proactive responses to negative narratives. By taking a strategic approach to media engagement, the radiation oncology community can help safeguard the discipline’s reputation, ensure that patients receive accurate information, and promote the benefits of modern RT as an integral part of cancer treatment [15].

It is of great importance to establish collaboration with the media to disseminate the positive results of treatments to the public and to dispel any false myths that may still exist on radiations and cancer care in general [16].

One might assume that the increased interest in RT in recent years would provide a much-needed boost to this specialty, as well as encouraging more trainees to enter the field. However, for this to occur, the media must also keep up to date with the latest developments in technology and the expanding applications of radiotherapy. Nevertheless, for these initiatives to have a meaningful impact, they must be complemented by other efforts and supported by additional initiatives for which scientific societies and professionals must be in charge [17].

Highlighting patient cures, innovative therapies and new treatment options helps ensure accurate information and enables radiation oncologists to become trusted sources in the public debate about our discipline.

One potential limitation of this study is its focus on a single traditional newspaper, Corriere della Sera, which, although influential, may not fully represent the current landscape of information dissemination. It seems likely that digital platforms, social media, and science journalism may well be playing an increasingly important role in shaping public and professional perceptions of radiation oncology, especially among younger generations.

Furthermore, the visibility of an article (e.g., front page vs. minor column) was not evaluated, although it can significantly influence its impact on the public.

## Conclusions

Italian media sentiment on radiotherapy (RT) has shifted over time, with increasing negativity and fewer positive themes, potentially may affecting public perceptions of RT’s efficacy and safety. Despite its crucial role in cancer treatment, media portrayal may influence opinions, harming patient access to necessary care. Another critical issue is the shortage of radiotherapy trainees, posing a threat to future healthcare quality. Addressing this challenge requires coordinated efforts from academic institutions, scientific societies, and policymakers to ensure the sustainability of radiotherapy and maintain high-quality cancer care for patients.

## References

[1] Beaton L, Bandula S, Gaze MN, Sharma RA (2019) How rapid advances in imaging are defining the future of precision radiation oncology. Br J Cancer 120(8):779–790 30911090 10.1038/s41416-019-0412-y PMC6474267

[2] Borras JM, Barton M, Grau C, Corral J, Verhoeven R, Lemmens V et al (2015) The impact of cancer incidence and stage on optimal utilization of radiotherapy: methodology of a population based analysis by the ESTRO-HERO project. Radiother Oncol 116(1):45–50 26002304 10.1016/j.radonc.2015.04.021

[3] Deng J, Feng Y, Ma C, Yin FF (2016) Novel technologies for improved treatment outcome and patient safety in cancer radiotherapy. Biomed Res Int 2016:3016454 27034929 10.1155/2016/3016454 PMC4791494

[4] Nichols AC, Theurer J, Prisman E, Read N, Berthelet E, Tran E et al (2022) Randomized trial of radiotherapy versus transoral robotic surgery for oropharyngeal squamous cell carcinoma: long-term results of the ORATOR trial. J Clin Oncol 40(8):866–875 34995124 10.1200/JCO.21.01961

[5] van As N, Yasar B, Griffin C, Patel J, Tree AC, Ostler P et al (2024) Radical prostatectomy versus stereotactic radiotherapy for clinically localised prostate cancer: results of the PACE-A randomised trial. Eur Urol 86(6):566–576 39266383 10.1016/j.eururo.2024.08.030

[6] Kurtz MP, MacDougall RD, Nelson CP (2018) Urology mythbusters: radiation and radiophobia. J Pediatr Urol 14(3):291–295 29571659 10.1016/j.jpurol.2018.01.022

[7] Im H, Huh J (2017) Does health information in mass media help or hurt patients? Investigation of potential negative influence of mass media health information on patients’ beliefs and medication regimen adherence. J Health Commun 22(3):214–222 28248627 10.1080/10810730.2016.1261970

[8] Wawrzuta D, Klejdysz J, Chojnacka M (2024) The rise of negative portrayals of radiation oncology: a textual analysis of media news. Radiother Oncol 190:110008 37972739 10.1016/j.radonc.2023.110008

[9] Gagliardi F, D’Ippolito E, Grassi R, Sangiovanni A, Menditti VS, Rubini D et al (2025) Being a radiation oncologist: times of crisis for European graduates. BJR|Open. 10.1093/bjro/tzaf016 40626057 10.1093/bjro/tzaf016 PMC12233087

[10] Young ME, Norman GR, Humphreys KR (2008) Medicine in the popular press: the influence of the media on perceptions of disease. PLoS ONE 3(10):e3552 18958167 10.1371/journal.pone.0003552 PMC2569209

[11] Grilli R, Ramsay C, Minozzi S (2002) Mass media interventions: effects on health services utilisation. Cochrane Database Syst Rev 1:Cd00038910.1002/14651858.CD00038911869574

[12] Kugler T, Kang KK, Kugler J, Arbetman-Rabinowitz M, Thomas J (2013) Demographic and economic consequences of conflict1. Int Stud Q 57(1):1–12

[13] Nardone V, D’Ippolito E, Grassi R, Sangiovanni A, Gagliardi F, De Marco G, Cappabianca S (2022) Non-oncological radiotherapy: a review of modern approaches. J Personal Med 12(10):1677 10.3390/jpm12101677 PMC9605240 36294816

[14] Citrin DE (2017) Recent developments in radiotherapy. N Engl J Med 377(11):1065–1075 28902591 10.1056/NEJMra1608986

[15] Wakefield MA, Loken B, Hornik RC (2010) Use of mass media campaigns to change health behaviour. Lancet 376(9748):1261–1271 20933263 10.1016/S0140-6736(10)60809-4 PMC4248563

[16] https://www.corriere.it/salute/sportello_cancro/cards/radioterapia-curare-tumori-10-falsi-miti-piu-diffusi-pazienti-familiari/cura-di-serie-b_principale.shtml. https://www.corriere.it/salute/sportello_cancro/cards/radioterapia-curare-tumori-10-falsi-miti-piu-diffusi-pazienti-familiari/cura-di-serie-b_principale.shtml.

[17] Lievens Y, Ricardi U, Poortmans P, Verellen D, Gasparotto C, Verfaillie C et al (2019) Radiation oncology. Optimal health for all, together. ESTRO vision, 2030. Radiother Oncol 136:86–97 31015134 10.1016/j.radonc.2019.03.031

